# A Review of the Therapeutic Potential of Recently Developed G Protein-Biased Kappa Agonists

**DOI:** 10.3389/fphar.2019.00407

**Published:** 2019-04-17

**Authors:** Kendall L. Mores, Benjamin R. Cummins, Robert J. Cassell, Richard M. van Rijn

**Affiliations:** ^1^Department of Medicinal Chemistry and Molecular Pharmacology, College of Pharmacy, West Lafayette, IN, United States; ^2^Department of Chemistry, College of Science, West Lafayette, IN, United States; ^3^Purdue Institute for Drug Discovery, West Lafayette, IN, United States; ^4^Purdue Institute for Integrative Neuroscience, West Lafayette, IN, United States

**Keywords:** kappa opioid receptor, beta-arrestin, G protein, signaling bias, nalfurafine, diphenethylamine, antinociception, side effects

## Abstract

Between 2000 and 2005 several studies revealed that morphine is more potent and exhibits fewer side effects in beta-arrestin 2 knockout mice. These findings spurred efforts to develop opioids that signal primarily via G protein activation and do not, or only very weakly, recruit beta-arrestin. Development of such molecules targeting the mu opioid receptor initially outpaced those targeting the kappa, delta and nociceptin opioid receptors, with the G protein-biased mu opioid agonist oliceridine/TRV130 having completed phase III clinical trials with improved therapeutic window to treat moderate-to-severe acute pain. Recently however, there has been a sharp increase in the development of novel G protein-biased kappa agonists. It is hypothesized that G protein-biased kappa agonists can reduce pain and itch, but exhibit fewer side effects, such as anhedonia and psychosis, that have thus far limited the clinical development of unbiased kappa opioid agonists. Here we summarize recently discovered G protein-biased kappa agonists, comparing structures, degree of signal bias and preclinical effects. We specifically reviewed nalfurafine, 22-thiocyanatosalvinorin A (RB-64), mesyl-salvinorin B, 2-(4-(furan-2-ylmethyl)-5-((4-methyl-3-(trifluoromethyl)benzyl)thio)-4H-1,2,4-triazol-3-yl)pyridine (triazole 1.1), 3-(2-((cyclopropylmethyl)(phenethyl)amino)ethyl)phenol (HS666), *N-n*-butyl-*N*-phenylethyl-*N*-3-hydroxyphenylethyl-amine (compound 5/BPHA), 6-guanidinonaltrindole (6′GNTI), and collybolide. These agonists encompass a variety of chemical scaffolds and range in both their potency and efficacy in terms of G protein signaling and beta-arrestin recruitment. Thus unsurprisingly, the behavioral responses reported for these agonists are not uniform. Yet, it is our conclusion that the kappa opioid field will benefit tremendously from future studies that compare several biased agonists and correlate the degree of signaling bias to a particular pharmacological response.

## Development of Signal-Biased Opioids in Search of Enhanced Therapeutic Windows

The majority of clinically used opioids selectively target the μ opioid receptor (μOR). Their use however, particularly in patients with chronic pain disorders, is complicated by side effects including opioid dependence, tolerance, constipation, itch and respiratory depression (Chou et al., 2009). The beginning of the 21^st^ century saw the emergence of the hypothesis that the side effect profile of μOR based drugs may be attributed to β-arrestin 2 signaling, as preclinical studies showed that mice lacking this protein displayed reduced morphine tolerance and respiratory depression (Bohn et al., 1999, 2000; Raehal et al., 2005). Despite morphine being already a relatively low efficacious β-arrestin 2 recruiter (Whistler and von Zastrow, 1998), the β-arrestin 2 KO mice studies were the driving factor for the development of so-called G protein-biased μOR agonists that preferentially signaled via the canonical G protein pathway, while further minimizing β-arrestin 2 recruitment and signaling. Such signal-biased opioids like TRV130 (Chen et al., 2013) and PZM21 indeed appeared to have improved therapeutic windows (Soergel et al., 2014; Manglik et al., 2016), and TRV130 advanced through all three clinical trial phases under the brand-name Olinvo®(oliceridine) for the treatment of moderate-to-severe pain via intravenous injection for example following abdominoplasty (Singla et al., 2017). However, recent preclinical studies have sowed doubt regarding the potential for these G protein-biased μOR agonists to reduce side effects like constipation, respiratory depression and dependence (Altarifi et al., 2017; Austin Zamarripa et al., 2018; Hill et al., 2018; Kliewer et al., 2019). Moreover, in October of 2018, the Food and Drug Administration (FDA) decided on a 8–7 vote not to approve Olinvo®, as the committee still had doubts as to whether the benefits associated with the drug outweighed the risks.

## Clinical Utility of Kappa Opioid Receptor (κOr) Selective Drugs

**FIGURE 1:**
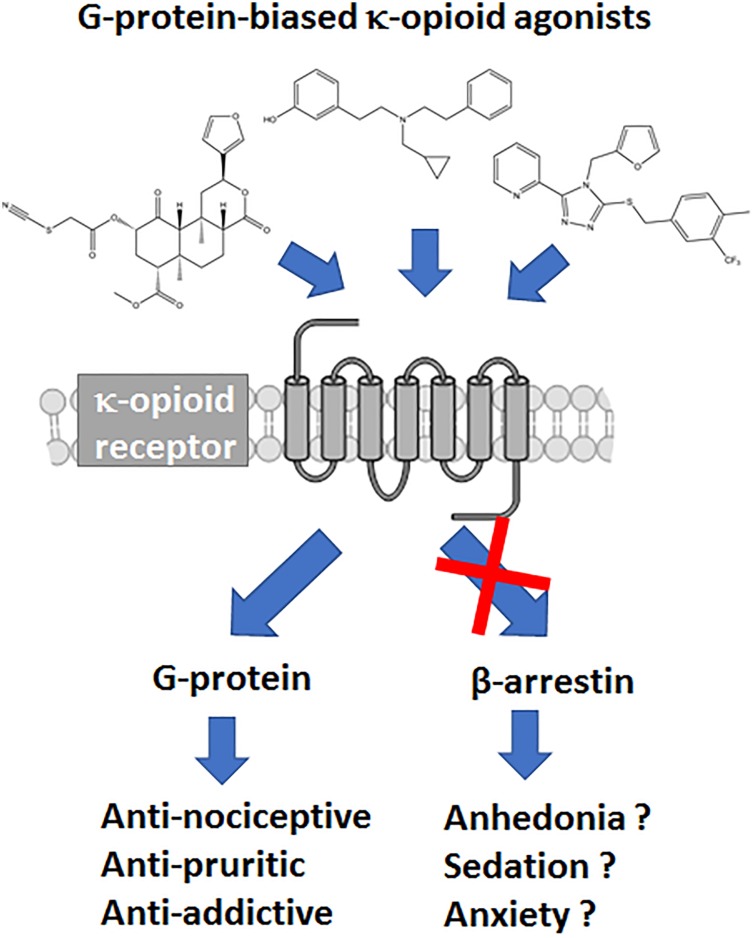
Hypothesized benefits of G protein-biased κ opioid receptor agonists.

The μOR is not the only opioid receptor modulating nociceptive transmission; the κ-opioid receptor (κOR) is a ubiquitously expressed G protein-coupled receptor (GPCR) whose signaling is involved in a wide range of biological processes, including nociception, stress, anxiety, depression, and substance use disorder (Al-Hasani and Bruchas, 2011; Chavkin, 2011; Bruchas and Roth, 2016). Whereas, μOR agonists like morphine are known to induce itch, κOR/dynorphin system has been linked with reducing pruritis as potential therapeutic action (Kardon et al., 2014; Cowan et al., 2015). However, compared to μOR and δ opioid receptor (δOR), the κOR/dynorphin system is more heavily associated with negative affect and stress responses of drug use (Chavkin and Koob, 2016). Therefore from a drug development point of view antagonism of κOR has received most attention, with κOR antagonists relieving depression-like and anxiety-like behaviors, attenuate stress responses and alcohol and cocaine use (Butelman et al., 2012; Walker et al., 2012; Karkhanis et al., 2017). Negative affect is an important factor in chronic pain management and the amygdala plays an important role in the circuitry associated with negative affect (Corder et al., 2019). Like μOR, activation of κOR produces analgesia, however the κOR/dynorphin system is heavily present in the amygdala (Land et al., 2008; Knoll et al., 2011; Kissler et al., 2014; Crowley et al., 2016). Thus there is a therapeutic promise for utilizing κORs in chronic pain settings, yet this requires producing κOR agonists with optimized pharmacological properties to ensure the drug produces analgesia, but are capable of mitigating the negative affect. Currently, the therapeutic potential of κOR agonists is limited by negative side effects they can produce, which include sedation, motor incoordination and dysphoria (or aversion in rodents) and psychotomimesis, the latter two effects being specific to κOR (Pfeiffer et al., 1986; Dykstra et al., 1987; Roth et al., 2002; Land et al., 2009) (Figure 1). The FDA has approved several non-selective opioids that target both the μOR and the κOR. However, these drugs act either as partial agonists (nalbuphine, nalmefene, pentazocine, butorphanol) or antagonists (buprenorphine) at the κOR, thus largely avoiding the side effects associated with strong κOR activation. Yet beyond partial agonism, an additional strategy may include biasing the κOR agonists signaling to a specific downstream pathway.

## Can Specifically Targeting G Protein-Biased Signaling Lead to the Development of Clinically Effective, κOR-Selective, Full Agonists?

**FIGURE 2:**
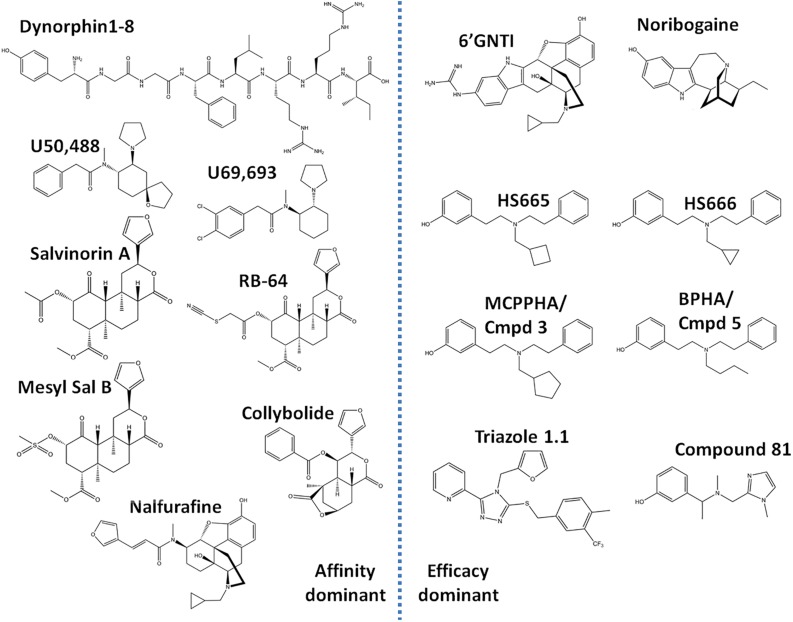
Chemical structures of ‘affinity-dominant’ and ‘efficacy-dominant’ G protein-biased κ opioid receptor agonists.

Similar to studies of μOR signaling bias, studies investigating κOR signaling have indicated that some of the negative side effects, such as aversion, could be mediated by β-arrestin 2 (Bruchas and Chavkin, 2010). Specifically, Bruchas et al. (2007), first revealed that U50,488 induced aversion requires p38 activation, which largely depends on G protein receptor kinase 3, which has been linked to β-arrestin 2 recruitment (Bruchas et al., 2006). In a follow up study, mice virally expressing the S369A κOR mutant, which does not get phosphorylated by G protein receptor kinase 3, in the dorsal raphe nucleus neurons projecting to the nucleus accumbens did not show U50,488 conditioned place aversion (CPA) (Land et al., 2009). Importantly, both antinociceptive and anti-pruritic efficacy of κOR agonists are retained in β-arrestin 2 knockout mice (Morgenweck et al., 2015; White et al., 2015) suggesting that G protein signaling is key for those beneficial effects. To harness the therapeutic potential of κOR activation, there has been an escalated search for κOR agonists which favor G protein signaling over β-arrestin recruitment. In this review, we provide a summary of the cellular bias and behavioral profiles of a dozen recently discovered G protein-biased κOR agonists (Figure 2), with the goal of discovering patterns or correlations between bias and specific adverse effects. This is particularly important as a study in 2015 found that κOR agonists-mediated aversion did not depend on β-arrestin 2 (White et al., 2015). In contrast to the earlier studies which indirectly associated β-arrestin 2 to the aversive effects, this particular study utilized β-arrestin 2 knockout mice. This study thus introduces some counterweight to the hypothesis that G protein-biased κOR agonists will produce fewer adverse effects. We will describe this controversy in more detail in the discussion.

## Methodology and Limitations of Measuring Biased Signaling *In Vitro*

The cellular environment strongly impacts the efficacy with which an agonist can engage a signal transduction pathway. For example strong expression/activity of regulators of G protein signaling will dampen G protein efficacy, whereas strong expression of G protein receptor kinases will enhance potency and efficacy of β-arrestin recruitment (Miess et al., 2018). Similarly, G-protein signaling can be measured at multiple levels, e.g., at the level of GTP exchange (GTPγS), at the level of second messenger production (e.g., cAMP) or at the level of gene transcription, resulting in different levels of signal amplification. Given the influence of cell environment and choice of readout it is important to be aware of limitations of detecting G protein or β-arrestin signaling. Therefore a lack of signaling is not evidence of antagonism or an exclusive preference for a single transduction pathway (Kenakin, 2015). To provide a strong measure of ligand bias it is recommended that G protein signaling and β-arrestin recruitment are measured in the same cell (Luttrell et al., 2015).

For the κOR agonists discussed here, the predominant assay for G protein signaling utilized [^35^S]GTPγS, whereas β-arrestin recruitment was primarily assessed using the proprietary PathHunter cells from DiscoverX. Although G protein signaling has also been assessed by measuring inhibition of cAMP (using a cAMP biosensor) and β-arrestin recruitment has been assessed using a TANGO assay and using bioluminescence resonance energy transfer (BRET). A 2014 study showed similarities and differences in potency and efficacy for κOR agonists when assessed in the TANGO or BRET assay, with the primary difference that partial agonism was more apparent in the TANGO assay. This however is not a rule, as partial agonism of β-arrestin recruitment at dopamine D_2_ receptor was more apparent using the PathHunter and BRET assay than using the TANGO assay (Allen et al., 2011).

## Calculations of Bias

The ability of agonists to engage multiple independent signal transduction routes means that one cannot rely on potency rank-order to compare signaling preference. Instead new methodologies were introduced to calculate a bias factor for agonists, which is a score for an agonist to preferentially signal via one pathway over another, relative to a reference compound. The endogenous κOR opioid dynorphin, the natural occurring opioid salvinorin A, and the synthetic κOR selective agonists U50,488 and U69,593 (Figure 2) are relatively unbiased, acting as equipotent and fully efficacious agonists for both G protein signaling and β-arrestin recruitment. Because of their unbiased nature, these κOR opioids often serve as a standard reference compound to assess the signaling bias for novel κOR agonists. The choice of reference compound across the studies discussed here was not consistent as some groups chose U50,488, while others used U69,593, or salvinorin A. The two most commonly used methods to calculate bias are the operational model/transduction coefficient method, and the equiactive method (Black and Leff, 1983; Rajagopal et al., 2011; Kenakin et al., 2012). The equiactive method, requires agonist responses to exhibit a perfect hill-slope, but can be quickly calculated solely using the EC_50_ and E_max_ values, however it does not account for receptor reserve (Rajagopal et al., 2011). A study on dopamine D_2_ agonists found little differences between these methods in calculated bias factor (Brust et al., 2015). The preferred method for the discussed papers in this review is the operational model/transduction coefficient method. A downside of both methods is that they work best when comparing full agonists, but fare worse when the agonist is a weak partial agonist in one of the assays. To optimize calculations the more data points (e.g., half-log dilution steps vs. full log step dilutions) that are used to create the dose response curve the more accurate the bias factor, this is particularly important for weak partial agonists, with a small signal-to-background window. To overcome the limitation of the partial agonist, it is advisable to concurrently run the cellular assay in competition mode; here the partial agonist is tested in the presence of a non-saturating concentration of the reference compound (Stahl et al., 2015; Dunn et al., 2018).

## G-Protein Biased Kappa Agonists

### Nalfurafine

17-Cyclopropylmethyl-3, 14 beta-dihydroxy-4,5 alpha-epoxy-6 beta-[*N*-methyl-*trans-*3-(3-furyl) acrylamido]morphinan hydrochloride (TRK-820) was discovered in 1998 in Japan in the lab of Dr. Hiroshi Nagase and found to produce antinociception without aversion (Nagase et al., 1998) and act as a selective κOR agonist (Seki et al., 1999). In 2002, TRK-820 was reported to reduce pruritis (Togashi et al., 2002), and is currently marketed as nalfurafine hydrochloride (Remitch®) in Japan as an antipruritic. Nalfurafine is the first, and currently only, κOR-selective agonist to have been approved for clinical use (Kumagai et al., 2010). Using early stage ERK phosphorylation as a proxy for G protein signaling and p38 phosphorylation as a measure for β-arrestin mediated signaling, nalfurafine was found to act as a G protein-biased agonist (Table 1) with a bias factor (Kenakin and Christopoulos, 2013; van der Westhuizen et al., 2014) of 7 at the rat κOR and 300 at the human κOR (Schattauer et al., 2017) relative to U50,488. It should be noted that MAPK activation is not a great endpoint to assess signal bias (Lovell et al., 2015). In a later study nalfurafine β-arrestin 2 recruitment was assessed in HEK293 cells using a galactosidase-based assay, which is similar to the PathHunter assay with a potency of 1.4 nM and efficacy of 129% relative to U50,488 (Liu et al., 2019). In CHO cells, G protein signaling as measured by [^35^S]GTPγS for nalfurafine is 0.025 nM (Fujij et al., 2012), while in mouse neuro2A cells the potency of nalfurafine, measured by GTPγS is lower at 0.11 nM (Liu et al., 2019) which would suggest nalfurafine is only slightly G protein-biased, with the caveat that the GTPγS was not measured in the HEK 293 cells used to measure β-arrestin recruitment. Preclinical behavioral studies in male C57BL/6 and CD-1 mice found that nalfurafine attenuated 5′-GNTI-induced scratching and produced antinociception in the warm water tail withdrawal assay. These behavioral effects were mediated by the κOR as they were blocked by the administration of the κOR antagonist norbinaltorphimine, and absent in κOR knockout mice (Inan et al., 2009; Schattauer et al., 2017; Liu et al., 2019). Additionally, in CD-1 mice, nalfurafine was not aversive up to a dose of 20 μg/kg, as measured in the CPA test. The same tested doses of nalfurafine did not cause anhedonia as evident from a lack in change in baseline intracranial self-stimulation (ICSS) threshold. Nalfurafine produced mild locomotor incoordination in the rotarod assay compared to U50,488 (Liu et al., 2019). Reportedly, nalfurafine only produced sedation at doses much higher (ED_50_ = 27 μg/kg) than needed to produce antinociception (ED_50_ = 3.3 μg/kg) (Endoh et al., 1999). In contrast to nalfurafine, U50,488 (0.5 mg/kg and higher) caused anhedonia and CPA (Liu et al., 2019). In Fisher 344 rats, a low dose of nalfurafine did not induce CPA, but reduced cocaine conditioned placed preference (CPP) (Mori et al., 2002) as well as morphine CPP (Tsuji et al., 2001).

**TABLE 1:** Overview of potency and efficacy of unbiased and G protein biased κOR agonists at human κOR for G protein and β-arrestin coupling, and of behavioral responses induced by the agonists.

Compound	G protein EC_50_ (nM)	Efficacy (%)	β-Arrestin2 EC_50_ (nM)	Efficacy (%)	Antinociception	Anti-itch	Incoordination/ sedation	Aversion	Anhedonia	Anxiety	Depression	References
U69,593	4–77	100–114	59–410	92–100	Y	–	Y	Y	Y	–	Y	Spetea et al., 2012, 2017; Schmid et al., 2013; Zhou L. et al., 2013; White et al., 2014, 2015; Lovell et al., 2015; Brust et al., 2016; Dunn et al., 2018; Ho et al.,2018
U50,488	1.5–24	93–100	36–1000	100–120	Y	Y	Y	Y	Y	B		Wang et al., 2008, 2016; Spetea et al., 2012; White et al., 2014; Brust et al., 2016; Dunn et al., 2018; Kivell et al., 2018; Liu et al.,2019
Sal A	4.5–40	100–120	28–249	77–95	Y	N	P	Y	Y	Y	Y	Roth et al., 2002; Harding et al., 2005; Zhang et al., 2005; Ansonoff et al., 2006; Carlezon et al., 2006; McCurdy et al., 2006; Wang et al., 2008; Sufka et al., 2014; White et al., 2014, 2015; Salaga et al., 2015; Kivell et al.,2018
Mesyl sal B	30	112	236	90	P	–	N	N	N	N	Y	Harding et al., 2005; Simonson et al., 2015; Kivell et al.,2018
RB-64*	0.077	95	391–1130^¶^	104–126	Y	–	N	Y	N	–	–	Yan et al., 2009; White et al.,2014, 2015
6-GNTI	2.1	37	ND-5.9	0–12	Y	–	N	N	–	–	–	Sharma et al., 2001; Waldhoer et al., 2005; Rives et al., 2012; Schmid et al., 2013; White et al., 2014; Zangrandi et al.,2016
HS665	1.8–5	88–110	380–463	30–55	Y	–	N-P	Y	–	–	–	Spetea et al., 2012, 2017; Guerrieri et al., 2016; Erli et al., 2017; Dunn et al.,2018
HS666	35–36	50–53	449	24	Y	–	N	N	–	–	–	Spetea et al., 2012, 2017; Guerrieri et al., 2016; Erli et al.,2017
Cmpd 5	4.7–46	46–94	ND	0	Y	–	N	–	–	–	–	Spetea et al., 2012; Erli et al., 2017; Dunn et al.,2018
Cmpd 3	0.6–3.9	83–100	720	55	Y	–	P	–	–	–	–	Spetea et al., 2012; Erli et al., 2017; Dunn et al.,2018
Nalfurafine	0.025–0.11	111	1.4–5.1	84–129	Y	Y	P	N	N	–	–	Nagase et al., 1998; Endoh et al., 1999; Seki et al., 1999; Tsuji et al., 2001; Mori et al., 2002; Togashi et al., 2002; Wang et al., 2005; Inan et al., 2009; Schattauer et al., 2017; Liu et al.,2019
Collybolide	9	22	NT	NT	Y	Y	N	Y	–	Y	–	Gupta et al.,2016
Triazole1.1	31–96	100	3338–8721	56–98	Y	Y	N	–	N	–	–	Zhou L. et al., 2013; Lovell et al., 2015; Brust et al., 2016; Ho et al.,2018
Noribogaine	8749	72	110	13	–	–	–	N	–	–	–	Maillet et al., 2015; Mash et al.,2016
Cmpd 81	530	∼75	8100	∼25	–	–	–	–	–	–	–	Zheng et al.,2017

Cellular characterization of potencies (50% effective concentration-EC_50_) for agonists at human κOR (*RB-64 at mouse κOR) for G protein signaling (GTPγS) and β-arrestin 2 recruitment (PathHunter). Agonist-mediated responses in behavioral assays were rated as positive (Y), negative (N) or partial (P), or bidirectional (B). NT, not tested; ND, not detectable. ^¶^ (Tango assay).

### RB-64

**FIGURE 3:**
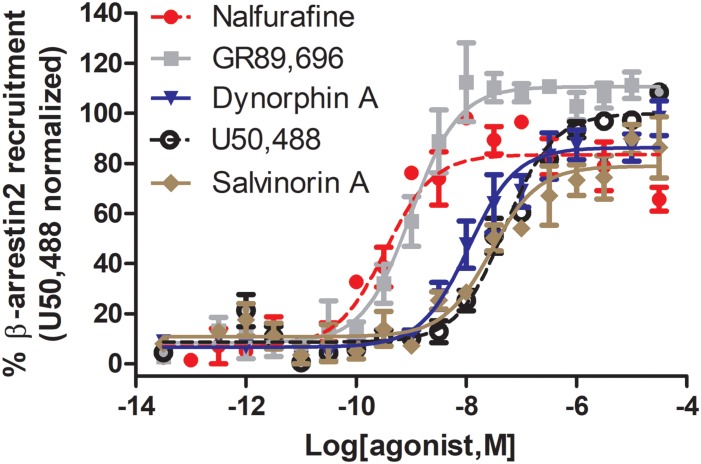
Nalfurafine and GR89,696, respectively potently and efficaciously recruit β-arrestin 2 recruitment following κOR activation. Dose-dependent recruitment of β-arrestin 2 to κOR following activation with nalfurafine (*n* = 3, red 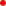), GR89,696 (*n* = 4, 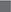), dynorphin A (*n* = 4, blue 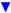), U50,488 (*n* = 4, ) and salvinorin A (*n* = 4, brown 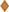) in PathHunter U2OS hOPRK1/β-arrestin-2 cells (DiscoverX, Fremont, CA, United States). Each concentration was tested in duplicate, and three independent dose-response curves were produced for each agonist as previously described (Chiang et al., 2016). Data is normalized to U50,488. Nalfurafine was purchased from AdooQ (Irvine, CA, United States) all other compounds were from Tocris (Minneapolis, MN, United States).

*Salvia divinorum* is a psychedelic plant that contains a non-nitrogenous diterpene, salvinorin A, with high κOR affinity (Roth et al., 2002). Depending on the technique and endpoint used, salvinorin A can be relatively unbiased (White et al., 2015) or G protein-biased (Kivell et al., 2018) (Figure 3). In male mice, salvinorin A is antinociceptive (Ansonoff et al., 2006; McCurdy et al., 2006) and was shown to non-significantly reduce scratching (Salaga et al., 2015), and induce aversion (Zhang et al., 2005; Sufka et al., 2014) and sedation (Zhang et al., 2005). The high affinity and selectivity of salvinorin A makes it an intriguing start point for the development G protein-biased κOR agonists. One such endeavor by the lab of Dr. Bryan Roth led to the development of RB-64 (22-thiocyanatosalvinorin A), a semi-synthetic structural derivative of salvinorin A (Yan et al., 2009). RB-64 was identified as a G protein-biased κOR agonist (Table 1) with a measured bias factor of 35–96 for G protein signaling relative to salvinorin A (White et al., 2014, 2015). However, this bias is purely driven by a 70–210 fold lower potency for RB-64 to recruit β-arrestin 2, as measured using the TANGO assay (Kroeze et al., 2015), compared to the potency to activate G protein signaling (cAMP GloSensor assay); in both assays RB-64 is a full agonist or even superagonist (Table 1). In both male and female C57BL/6 mice, RB-64 was shown to produce antinociception in the hot-plate assay to a similar degree as U69,593 and salvinorin A. However, in this study RB-64 was the only κOR agonist that did not produce locomotor incoordination in the rotarod assay (White et al., 2015). Surprisingly, based on the hypothesis that aversion/anhedonia is mediated by β-arrestin 2, RB-64, as well as salvinorin A and U69,593, produced CPA in both wild-type and β-arrestin 2 knockout mice (White et al., 2015), suggesting aversion is not mediated by β-arrestin 2. Given that G protein-biased μOR agonist have not consistently reproduced the observed phenotype of μOR agonists in β-arrestin 2 knockout mice, we should still be cautious in interpreting results from global knockout mice. The recently developed conditional β-arrestin 2 knockout mice (Urs et al., 2016) should be a more precise tool to study the role of β-arrestin 2 in CPA. RB-64 caused a weak right-ward shift in ICSS response rate, compared to a moderate shift by salvinorin A and a strong shift by U69,593, suggesting RB-64 did not induce pronounced anhedonia. It should be noted that sedation is a possible confound in interpreting the anhedonic effects of salvinorin A and U69,593 in this assay. The correlation between ICSS reduction and bias factor may indicate a role for β-arrestin 2 signaling in producing anhedonia (White et al., 2015).

### Mesyl Salvinorin B

((2S,4aR,6aR,7R,9S,10aS,10bR)-9-(methanesulfonyloxy)-2-(3-furanyl)dodecahydro-6a, 10b-dimethyl-4,10-dioxy-2H-naptho [2,1-c]pyran-7-carboxylic acid methyl ester) is a semi-synthetic derivative of salvinorin A (Harding et al., 2005), originating from the lab of Dr. Thomas Prisinzano, that has a G protein bias similar to that of RB-64, in that it is a full agonist for both G protein and β-arrestin 2 recruitment (Table 1), but >1000 fold more potent in the G protein activation assay (Kivell et al., 2018). Mesyl sal B was only weakly analgesic in the warm-water tail withdrawal test, but did reduce cocaine-induced hyperlocomotor activity and cocaine seeking behavior (Simonson et al., 2015; Kivell et al., 2018). In male Sprague-Dawley male rats mesyl sal B did not produce CPA or anxiety-like behavior nor did it reduce sucrose-reinforced responding, a measure of anhedonia, although it did increase depression-like behavior in the forced swim test (Kivell et al., 2018). In B6.SJL male mice, Mesyl sal B did not impact rotarod performance (Kivell et al., 2018).

### Triazole 1.1

In 2012, a team of researchers from Duke University, the Scripps Research Institute, Sanford-Burnham Medical Research Institute and Kansas University employed a high-throughput strategy utilizing κOR β-arrestin 2 PathHunter cells to screen nearly 300,000 compounds to identify novel κOR agonists and antagonists (Frankowski et al., 2012). The screen identified novel antagonist and agonist chemotypes including triazole 1.1 (Frankowski et al., 2012), which was shown to be biased toward G protein coupling relative to U69,593 as determined by [^35^S]GTPγS and PathHunter β-arrestin 2 recruitment assays (Table 1) and produce antinociception in male C57BL/6 mice in the warm-water tail immersion test (Zhou L. et al., 2013; Lovell et al., 2015). A more detailed analysis of the behavioral effects of triazole 1.1 revealed that the biased κOR agonist also reduced chloroquine-mediated scratching, but did not reduce ambulatory locomotion at analgesic doses (Brust et al., 2016). Unlike the unbiased κOR agonist U50,488, triazole 1.1 did not strongly reduce dopamine release in the nucleus accumbens, which could indicate that triazole 1.1 will not produce dysphoria/aversion. In line with these findings, U50,488, but not triazole 1.1, produced a rightward shift in ICSS in rats, suggesting unlike U50,488 triazole 1.1 did not produce anhedonia (Brust et al., 2016). It should be noted that in this study the positive control U50,488 decreased the maximum response rate, which may imply sedation and confound data interpretation.

### Diphenethylamines

In 2012, first diphenethylamine derivatives with κOR activity and antinociceptive efficacy created by the lab of Drs. Mariana Spetea/Helmut Schmidhammer were reported (Spetea et al., 2012). In a follow up study, Spetea et al. (2017) used [^35^S]GTPγS and the PathHunter β-arrestin 2 recruitment assays to determine if HS665 and HS666 exhibit bias toward the activation of G protein over β−arrestin 2−mediated signaling (Table 1), when compared with U69,593; the bias factors for HS665 and HS666 were determined to be 389 and 62, respectively. In a warm-water tail withdrawal assay HS665 and HS666, administered intracerebroventricularly, dose−dependently produced antinociception in male C57BL/6 wild-type mice, but not in κOR knockout mice. Neither HS665 nor HS666 produced locomotor coordination issues as measured in the rotarod assay, however only HS665, but not HS666 induced CPA (Spetea et al., 2017). When injected systemically (intraperitoneally) HS665 (although named MCBPHA in this paper) produced modest motor incoordination in male C57BL/6 mice in the rotarod assay, which was similar to MCPPHA (which was compound 3 in Spetea et al., 2012), but less than U50,488 (Dunn et al., 2018). A library of derivatives of HS665 and HS665 were synthesized with several compounds showing subnanomolar affinity and exceptional κOR selectivity, as well as high G-protein potency acting as either full or partial agonists (Erli et al., 2017). While those new derivatives were not assessed for β-arrestin recruitment, the new derivatives displayed increased antinociceptive potencies compared with U50,488, HS665 and HS666 in the acetic acid-induced writhing test (Erli et al., 2017). Another derivative of the trialkylamine scaffold, BPHA (compound 5 in Spetea et al., 2012), did not measurably recruit β-arrestin 2 as determined using the PathHunter cell assay using U50,488 as reference compound (Table 1) and did not cause locomotor incoordination (Dunn et al., 2018). These results appear to indicate a correlation between bias factor or β-arrestin 2 recruitment efficacy and κOR agonist-induced rotarod incoordination.

### 6′-GNTI

After its initial synthesis in 2001 in the lab of Dr. Philip Portoghese (Sharma et al., 2001), the κOR agonist 6′-guanidinonaltrindole (6′-GNTI) was deemed to mediate antinociception through interacting specifically with heteromers of the κOR and δOR in a publication by Waldhoer et al. (2005). In 2012, using BRET assays, Rives et al. (2012) found that 6′-GNTI is a partial G protein agonist (Table 1), with no detectable β-arrestin 2 recruitment. The κOR agonist GR89,696 has been suggested to also interact with κOR-δOR heteromers (Brissett et al., 2012), however, in contrast to 6′-GNTI, GR89,696 reportedly displays β-arrestin2 bias (White et al., 2014), giving pause to a hypothesis that the κOR-δOR heteromer adopts a G protein biased conformation. Similar to HS666, 6′GNTI was not significantly aversive in male C57BL/6 mice in the CPA paradigm, nor was it sedative (Zangrandi et al., 2016). Activation of the κOR in striatal neurons with 6′-GNTI led to phosphorylation of Akt, but not ERK1/2, which is in contrast to the unbiased κOR agonist U69,593 which induced phosphorylation of both kinases (Schmid et al., 2013). In striatal neurons from β-arrestin 2 knockout mice, persistent ERK activation by 6′-GNTI was β-arrestin 2-dependent, whereas Akt phosphorylation was pertussis toxin sensitive, indicative of a G_αi_-protein-mediated mechanism (Schmid et al., 2013). The chronology of the pharmacological characterization of 6′-GNTI mimics that of the δOR agonist TAN-67, which was suggested to act on δOR-μOR heteromers (van Rijn and Whistler, 2009), and was subsequently found to be G protein-biased (Chiang et al., 2016) *in vitro* and in mouse dorsal striatum (Robins et al., 2018).

### Collybolide

Collybolide is a natural product first extracted from the fungus *Collybia maculata* by the research group of Dr. Pierre Potier (Bui et al., 1974). Collybolide shares structural similarity, particularly a familiar furyl-δ-lactone core, with salvinorin A (Gupta et al., 2016). In male C57BL/6 mice, systemic (intraperitoneal) injection of collybolide produced antinociception in a tail-flick assay and reduced chloroquine-induced itch. Collybolide was not sedative at a dose that produced antinociception, but did produce aversion in a CPA paradigm and also produced mild, norbinaltorphimine reversible anxiety (Gupta et al., 2016). *In vitro*, collybolide was a potent but partial agonist in the [^35^S]GTPγS assay, though β-arrestin 2 recruitment was not tested (Table 1). However, compared to U69,593, collybolide preferentially induced phosphorylation of Akt over ERK (Gupta et al., 2016), which is a pharmacological profile also seen for the G protein-biased agonist 6′-GNTI.

### Low Potency G Protein-Biased κOR Agonists

A team of researchers from the Universities of North Carolina and Southern California utilized the crystal structure of the κOR, which was resolved in 2012 bound to κOR antagonist JDTic (Wu et al., 2012), to virtually screen five million fragment-like and lead-like compounds resulting in the identification of 11 hits subcategorized into four chemotypes (Zheng et al., 2017). Compound 81 (3-(1-(methyl((1-methyl-1H-imidazol-2-yl)methyl)amino)ethyl)phenol), a derivative of one of the hits (the balanced agonist compound 23), was determined to act as a G protein-biased κOR agonist (Table 1), using the cAMP GloSensor assay, but was found to only weakly recruit β-arrestin 2 using the TANGO assay, with a bias factor of 6 against salvinorin A (Zheng et al., 2017). Thus far, compound 81 has not been tested *in vivo*, perhaps in part because of its relatively low potency (530 nM). Structurally, compound 81 resembles the G protein-biased κOR agonists, HS666 and BPHA/compound 5 (Figure 2) and thus perhaps derivatization of compound 81 may yield a more potent G protein-biased κOR agonist that can be assessed in animals. Another low potency G protein-biased κOR agonist is noribogaine, which is an active metabolite of the psychoactive alkaloid ibogaine found in plants belonging to the Apocynaceae family such as *Tabernanthe iboga* (Davis et al., 2017; Malcolm et al., 2018; Mash et al., 2018). In [^35^S]GTPγS binding assays, noribogaine displayed partial κOR agonist activity with an E_Max_ of 72% relative to U69,593 (Table 1). In the DiscoverX PathHunter β-arrestin 2 recruitment assay, noribogaine was a very weak recruiter (E_Max_ of 13% normalized to U69,593). No significant CPP or CPA was observed for 10, 30, and 100 mg/kg (oral) noribogaine (Mash et al., 2016), although given the low potency of noribogaine κORs may not play a major role in any observed behavior at these doses.

## Conclusion and Future Directions

G protein bias may either be affinity/potency-dominant or efficacy-dominant (Table 1). A potential concern is that, despite using the same cellular assays, variations in agonist potency were determined by different, or even the same labs, potentially due to differences in expression levels of the receptor and signaling proteins that occurred during cell passaging. For example the reported arrestin recruitment potency for U69,593 has been reported to be as low as 67.7 nM (Spetea et al., 2017) and as high as 410 nM (Dunn et al., 2018), similarly for U50,488 potency has ranged from 36 to 1000 nM (Dunn et al., 2018; Liu et al., 2019). In our hands the values were slightly different as well, finding the following potencies and efficacies for U50,488 (EC_50_ = 51.5 ± 12, α = 100 ± 1), nalfurafine (EC_50_ (nM) = 5.19 ± 1.3, α = 84 ± 7), and salvinorin A (EC_50_ = 27.5 ± 10, α = 76 ± 9), whilst also confirming super recruitment by GR89,696 (EC_50_ = 9.90 ± 3.6, α = 112 ± 3), but partial recruitment of dynorphin A (EC_50_ = 21.0 ± 13, α = 87 ± 5) relative to U50,488 (Figure 3). These potency differences as well as which reference compound is used will impact the calculated bias factor, which is the reason why we did not include bias factors in Table 1. It would be helpful for the field if the endogenous agonist dynorphin A was used as reference compound to ensure easier comparison between studies.

As detailed in Section “Can Specifically Targeting G Protein-Biased Signaling Lead to the Development of Clinically Effective, κOR-Selective, Full Agonists?,” cellular context strongly impacts the calculation of signaling bias. The majority of the discussed papers utilized U20S human bone osterosarcoma cells stable overexpressing β-arrestin2 and κOR, which most likely do not resemble the cellular context of, for example, striatal neurons, a region selectively and strongly expressing regulator of G protein signaling 9-2 (Gold et al., 1997; Rahman et al., 1999). Moreover, cellular context is known to change during a chronic pain state (Xiao et al., 2002; Obara et al., 2009; Zhou Y. et al., 2013). Unfortunately, many of the studies investigating the analgesic potency of the G protein-biased κOR agonists discussed here were conducted in naïve mice using acute/reflexive pain models that are not as translatable to patients with chronic pain. Native receptor expression and G protein signaling can be assessed in cultured neurons or cryopreserved brain sections using radioligand binding. However, the biggest hurdle currently is the lack of a radiolabel or biosensor to assess β-arrestin signaling in such cultures or brain sections, at least without exogenously introducing a genetic β-arrestin construct. If a peptide could be produced that can reach and bind to the intracellular side of GPCRs and that can be competitively displaced upon β-arrestin binding to the GPCR, such a peptide could be turned into a radiolabel to assess bias factor in neurons under native conditions.

**FIGURE 4:**
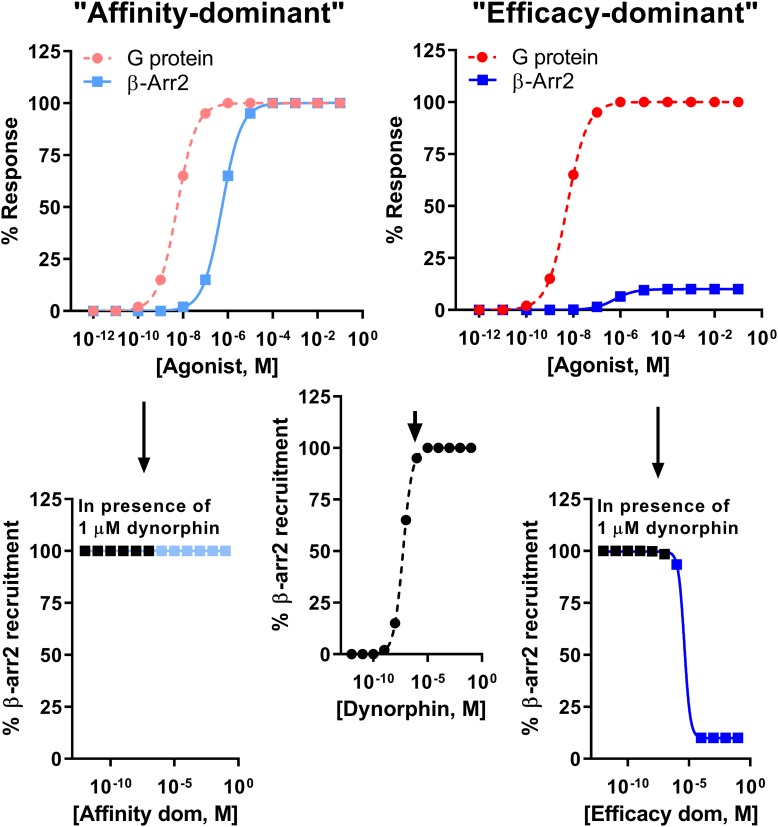
Difference between an “affinity-dominant” and “efficacy-dominant” G protein-biased agonist. An “affinity-dominant” κOR agonist has a higher affinity for the κOR conformation that activates G proteins than that recruits β-arrestin 2: see top left panel for an example of a κOR agonist that resembles RB-64, with a G protein-coupling EC_50_ potency of 5.5 nM (dotted line, pink 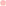, G-protein) and EC_50_ potency for β-arrestin 2 recruitment of 550 nM and 100% efficacy (solid line, light blue 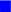). In contrast an ‘efficacy-dominant” κOR agonist, that resembles HS666 with an EC_50_ potency for β-arrestin 2 recruitment of 550 nM, but 10% efficacy (solid line, blue 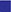), only weakly recruits β-arrestin 2 even at high concentrations (top right panel). Consider the endogenous agonist dynorphin A, which recruits β-arrestin 2 at 100% efficacy (middle panel). At high concentrations the affinity-dominant agonist will displace dynorphin from the κOR, yet retain highly efficacious recruitment of β-arrestin 2 (bottom left panel). In contrast the efficacy-dominant agonist will reduce β-arrestin 2 recruitment efficacy once this type of agonists displaces dynorphin from the κOR (bottom right) panel.

It is important to note the limitation of comparing bias factors of two G protein-biased agonists even if they are calculated using the same reference compound. Specifically two compounds may have the same bias factor, but have completely different β-arrestin recruitment efficacy [affinity-dominant vs. efficacy-dominant (Kenakin, 2015)]. The physiological difference would be that a weak/partial β-arrestin recruiter would serve as a functional antagonist for β-arrestin recruitment relative to the endogenous full agonist response (Kenakin, 2015) (Figure 4). For example, HS665 is a partial recruiter of β-arrestin 2 but has a higher calculated bias factor than HS666 and BPHA, which show minimal β-arrestin 2 recruitment. Yet, despite the lower bias factor it is HS666 and BPHA that do not produce locomotor incoordination. This is not too dissimilar to findings that β-arrestin 2 recruitment efficacy of δOR agonists was tightly correlated with modulation of alcohol use (Chiang et al., 2016).

The finding that κOR agonists regardless of signaling bias were still producing CPA in β-arrestin 2 knockout mice is disconcerting for the therapeutic promise of G protein-biased κOR agonists. A recent study by Liu et al. (2018) compared the phosphoproteome of the striatum (amongst other regions) following exposure to U50,488, HS665, RB-64, 6′-GNTI and HS666. Notably, compared to the arrestin recruiting agonists U50,488, HS665 and RB64, the G protein-biased 6′GNTI and HS666 did not activate mechanistic target of rapamycin (mTOR) signaling. Inhibition of mTOR abolished CPA induced by U50,488 thus linking the mTOR transduction pathway to this important side effect. Close examination of their results revealed that the weak β-arrestin 2 recruiting κOR agonists 6′-GNTI and HS666 often displayed unique modulation of protein phosphorylation relative to the more efficacious β-arrestin 2 recruiters (Liu et al., 2018). Thus, even if β-arrestin 2 is not involved in all these phosphorylation events/signaling cascades, searching for efficacy-dominant G protein-biased κOR agonists may provide the desired therapeutic window to treat pain with reduced side effects. In a follow up study, nalfurafine surprisingly, as it still efficaciously recruits β-arrestin 2, also did not activate mTOR nor induce CPA (Liu et al., 2019). Activation of mTOR however could not explain for κOR agonist-induced locomotor inhibition, but importantly mTOR also did not play a role in the antipruritic and antinociceptive effects of U50,488 (Liu et al., 2019). Thus, currently it is unclear what precise role β-arrestin 2 plays, if any, in mediating κOR agonist-mediated aversion and in general there does not yet seem to be a consistent consensus on the therapeutic and side-effect profile of affinity-dominant and efficacy-dominant G protein-biased κOR agonists.

After the initial antagonist-bound crystal structure of the κOR (Wu et al., 2012), two agonist-bound κOR structures have been produced, one in which the κOR was bound to the endogenous agonist dynorphin 1–13 (O’Connor et al., 2015) and the other bound to the unbiased agonist MP1104 (Varadi et al., 2015; Che et al., 2018). It would be incredibly insightful for the opioid field to study an opioid receptor bound to a signal-biased ligand. However, crystallizing ligand-bound structures requires the use of ligands that have very strong affinity for the receptor. The currently available agonist-bound structures may aid in the rational design of high affinity G protein-biased κOR agonists, which can then be used to crystallize a G protein-biased conformation of the κOR. The recent discovery of numerous G protein-biased κOR agonists has substantially expanded the pharmacological toolbox and perhaps one of the recently developed efficacy-dominant G protein-biased opioids, like HS665, may already have the correct properties to accomplish this goal. Hopefully, additional κOR biased agonists will be developed to strengthen the diversity of the current crop and they will be used in tandem to investigate the role of biased signaling in human (patho)physiology in more detail, including by studying downstream phosphorylation events. Accordingly, they can aid in the search for better, more efficacious therapies for disorders, such as chronic pain, and pruritis.

## Author Contributions

RvR supervised KM, BC, and RC. RC carried out the β-arrestin 2 recruitment experiments. KM, BC, and RvR wrote the first draft of the manuscript. KM, BC, RC, and RvR edited and proofread the manuscript and approved the final draft.

## Conflict of Interest Statement

The authors declare that the research was conducted in the absence of any commercial or financial relationships that could be construed as a potential conflict of interest.
